# Diagnostic Performance of Fluorine-18-Fluorodeoxyglucose Positron Emission Tomography in the Postchemotherapy Management of Patients with Seminoma: Systematic Review and Meta-Analysis

**DOI:** 10.1155/2014/852681

**Published:** 2014-05-15

**Authors:** Giorgio Treglia, Ramin Sadeghi, Salvatore Annunziata, Carmelo Caldarella, Francesco Bertagna, Luca Giovanella

**Affiliations:** ^1^Department of Nuclear Medicine and PET/CT Center, Oncology Institute of Southern Switzerland, Via Ospedale 12, 6500 Bellinzona, Switzerland; ^2^Nuclear Medicine Research Center, Mashhad University of Medical Sciences, Mashhad, Iran; ^3^Institute of Nuclear Medicine, Catholic University of the Sacred Heart, 00168 Rome, Italy; ^4^Department of Nuclear Medicine, University of Brescia and Spedali Civili, 25123 Brescia, Italy

## Abstract

*Objective*. To meta-analyze published data about the diagnostic performance of fluorine-18-Fluorodeoxyglucose (^18^F-FDG) positron emission tomography (PET) and PET/computed tomography (PET/CT) in the postchemotherapy management of patients with seminoma. *Methods*. A comprehensive literature search of studies published through January 2014 on this topic was performed. All retrieved studies were reviewed and qualitatively analyzed. Pooled sensitivity and specificity, positive and negative predictive values (PPV and NPV), accuracy, and area under the summary ROC curve (AUC) of ^18^F-FDG-PET or PET/CT on a per examination-based analysis were calculated. Subgroup analyses considering the size of residual/recurrent lesions were carried out. *Results*. Nine studies including 375 scans were selected. The pooled analysis provided the following results: sensitivity 78% (95% confidence interval (95% CI): 67–87%), specificity 86% (95% CI: 81–89%), PPV 58% (95% CI: 48–68%), NPV 94% (95% CI: 90–96%), and accuracy 84% (95% CI: 80–88%). The AUC was 0.90. A better diagnostic accuracy of ^18^F-FDG-PET or PET/CT in evaluating residual/recurrent lesions >3 cm compared to those <3 cm was found. *Conclusions*. ^18^F-FDG-PET and PET/CT were demonstrated to be accurate imaging methods in the postchemotherapy management of patients with seminoma; nevertheless possible sources of false-negative and false-positive results should be considered. The literature focusing on this setting still remains limited and cost-effectiveness analyses are warranted.

## 1. Introduction

Seminoma is a malignant germ cell tumor of the testis or, more rarely, of extragonadal locations which originates in the germinal epithelium of the seminiferous tubules. About half of germ cell tumors of the testis are seminomas. Metastatic seminoma is the paradigm of a curable cancer and the cure rate has now attained 95%, with extrapulmonary visceral metastases being the main identified prognostic factor [1, 2]. The optimal postchemotherapy management of patients with seminoma has been widely debated. In fact, postchemotherapy residual lesions at morphological imaging are frequent and surgical resection of these findings usually reveals necrosis or fibrosis [3]. Some centers suggested performing surgery for all residual lesions >3 cm because the likelihood of viable tumor increases in residual masses larger than 3 cm, while others suggested observing and using salvage treatment only if the lesions fail to shrink or for clearly documented relapse taking into account the technical difficulties and potential morbidity of surgery [3].

Over the past 2 decades, the postchemotherapy management of patients with seminoma has evolved due to the increasing use of fluorine-18 fluorodeoxyglucose positron emission tomography (^18^F-FDG-PET). This method alone or combined with computed tomography (PET/CT) has been proposed as noninvasive tool to assess the disease extent in cancer patients. Since ^18^F-FDG is a glucose analogue, this radiopharmaceutical may be very useful in detecting malignant lesions which usually present high glucose metabolism [4]. Hybrid PET/CT device allows enhanced detection and characterization of neoplastic lesions, by combining the functional data obtained by PET with morphological data obtained by CT [4]. The incorporation of noninvasive imaging modalities, such as ^18^F-FDG-PET or PET/CT, into the management algorithm may allow better delineation of the presence of viable residual tumor and thus allow better risk stratification in patients with seminoma [2, 3].

Several prospective and retrospective studies evaluated the diagnostic performance of ^18^F-FDG-PET or PET/CT in the postchemotherapy management of patients with seminoma reporting conflicting results. The aim of our study is to perform an updated systematic review and meta-analysis in order to provide more evidence-based data in this setting.

## 2. Methods

This systematic review and meta-analysis were performed according to the “Preferred Reporting Items for Systematic Reviews and Meta-Analyses” (PRISMA) statement which describes an evidence-based minimum set of items for reporting in systematic reviews and meta-analyses [5].

### 2.1. Search Strategy

A comprehensive computer literature search of the PubMed/MEDLINE and Scopus databases was conducted to find relevant published articles on the diagnostic performance of ^18^F-FDG-PET or PET/CT in the postchemotherapy management of patients with seminoma. We used a search algorithm that was based on a combination of the following terms: (a) “PET” or “positron emission tomography” and (b) “seminoma” or “seminomatous” or “testis” or “testicular” or “germinal” or “germ cell”. No beginning date limit was used; the search was updated until January 31, 2014. To expand our search, references of the retrieved articles were also screened for additional studies.

### 2.2. Study Selection

Studies or subsets in studies investigating the diagnostic performance of ^18^F-FDG-PET or PET/CT in the postchemotherapy management of patients with seminoma (including evaluation of residual masses after chemotherapy and restaging) were eligible for inclusion. The exclusion criteria were (a) articles not within the field of interest of this review; (b) review articles, editorials or letters, comments, and conference proceedings; (c) case reports or small case series (less than 10 patients with seminoma); (d) articles evaluating patients with seminoma at initial staging; (e) insufficient information to reassess sensitivity and specificity in the postchemotherapy management; (f) articles not in English language; (g) possible data overlap (in such cases the most complete article was included).

Three researchers independently reviewed the titles and abstracts of the retrieved articles, applying the inclusion and exclusion criteria mentioned above. Articles were rejected if they were clearly ineligible. The same three researchers then independently reviewed the full-text version of the remaining articles to determine their eligibility for inclusion. Disagreements were resolved in a consensus meeting.

### 2.3. Data Extraction

For each included study, information was collected concerning basic study (authors, journals and year of publication, country of origin, and study design), patient characteristics, and technical aspects (device used, radiopharmaceutical injected dose, time between ^18^F-FDG injection and image acquisition, image analysis, and applied reference standard). For each study the number of true-positive, false-positive, true-negative, and false-negative findings for ^18^F-FDG-PET or PET/CT was recorded on a per examination-based analysis considering the qualitative PET analysis (visual analysis) performed by the authors.

### 2.4. Quality Assessment

The 2011 Oxford Center for Evidence-Based Medicine checklist for diagnostic studies was used for quality assessment of the included studies [15]. This checklist has 5 major parts as follows: representative spectrum of the patients, consecutive patient recruitment, ascertainment of the gold standard regardless of the index test results, independent blind comparison between the gold standard and index test results, and enough explanation of the test to permit replication.

### 2.5. Statistical Analysis

Sensitivity, specificity, accuracy, positive and negative predictive value, positive and negative likelihood ratio (LR), and diagnostic odd ratio (DOR) of ^18^F-FDG-PET or PET/CT in the postchemotherapy management of patients with seminoma were obtained from individual studies on a per examination-based analysis. A random effects model was used for statistical pooling of the data. Pooled data were presented with 95% confidence intervals (95% CI). An *I* ^2^ index was used to test for heterogeneity among the studies. The area under the summary ROC curve (AUC) was calculated to measure the accuracy of ^18^F-FDG-PET or PET/CT. For publication bias evaluation, funnel plots, Egger's regression intercept [16], and Duval and Tweedie's method [17] were used. Subgroup analyses on the diagnostic performance of ^18^F-FDG-PET or PET/CT in patients with postchemotherapy residual/recurrent lesions at CT with size < or >3 cm were also carried out.

Statistical analyses were performed using Meta-DiSc statistical software version 1.4 (Unit of Clinical Biostatistics, Ramón y Cajal Hospital, Madrid, Spain) and Comprehensive Meta-Analysis (CMA) software version 2 (BioStat, Englewood, NJ, USA).

## 3. Results

### 3.1. Literature Search

**Figure 1:**
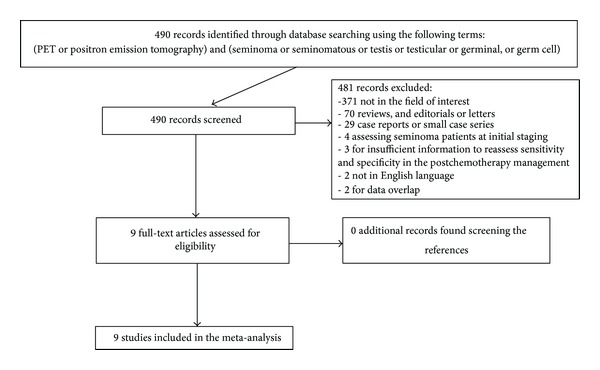
Flowchart of the search for eligible studies on the diagnostic performance of ^18^F-FDG-PET or PET/CT in the postchemotherapy management of patients with seminoma.

The comprehensive computer literature search from PubMed/MEDLINE and Scopus databases revealed 490 articles. Reviewing titles and abstracts, 481 articles were excluded: 371 were excluded because they were not in the field of interest of this review, 70 were excluded as reviews and editorials or letters, 29 were excluded as case reports or small case series, 4 were excluded as articles evaluating patients with seminoma at initial staging, 3 were excluded for insufficient information to reassess sensitivity and specificity in the postchemotherapy management [18–20], 2 articles were excluded because they were not in English language [21, 22], and 2 articles were excluded for possible data overlap [23, 24]. Finally, nine articles including 375 patients were selected and were eligible for the systematic review and meta-analysis [6–14]; no additional studies were found screening the references of these articles (Figure 1). The characteristics of the included studies are presented in Tables 1, 2, 3, and 4.

### 3.2. Qualitative Analysis (Systematic Review)

Using the database search, 9 original articles written over the past 15 years were selected [6–14]. About the study design, three of these studies were prospective [9, 11, 14], 5 were retrospective [6–8, 10, 13], and in one article this information was not provided [12]. Three studies were multicentric [7, 9, 11], whereas six were monocentric [6, 8, 10, 12–14]. Most of the patients performed ^18^F-FDG-PET or PET/CT for evaluating abdominal residual lesions at CT imaging after chemotherapy (Table 1).

Two studies used hybrid PET/CT [6, 8], whereas seven studies used PET only [7, 9–14]. Heterogeneous technical aspects between the included studies were found (Table 2). PET image analysis was performed by using qualitative criteria (visual analysis) in all the included studies [6–14] and adjunctive semiquantitative criteria (based on the calculation of the standardized uptake value (SUV)) in 3 out of 9 articles [9, 12, 14].

The reference standard used to validate the ^18^F-FDG-PET or PET/CT findings was quite different in the included studies (Table 4). The results of the quality assessment of the studies included in this meta-analysis, according to the 2011 Oxford Center for Evidence-Based Medicine checklist for diagnostic studies, are shown in Table 4.

Most of the studies included in this pooled analysis support the usefulness of ^18^F-FDG-PET or PET/CT in the postchemotherapy management of patients with seminoma compared to CT alone [6–13]. Abdominal residual lesions at CT are quite frequent in patients with seminoma after chemotherapy and conventional imaging methods often do not discriminate between residual neoplastic lesions or fibrotic tissue, whereas ^18^F-FDG-PET may provide complementary metabolic information on these lesions. Furthermore, ^18^F-FDG-PET may detect early recurrent disease in patients with seminoma and normal CT findings [6, 13], because functional abnormalities may precede morphological changes. On the other hand, possible sources of false-negative (small malignant lesions or with low proliferative index) and false-positive results (mainly inflammatory lesions) of ^18^F-FDG-PET or PET/CT in the postchemotherapy management of patients with seminoma should be kept in mind [6–14].

About the impact on the clinical management a recent study demonstrated that ^18^F-FDG-PET or PET/CT may provide valuable information to this regard, particularly for clinical surveillance and posttherapy assessment and when relapse is suspected [6].

### 3.3. Quantitative Analysis (Meta-Analysis)

**Figure 2:**
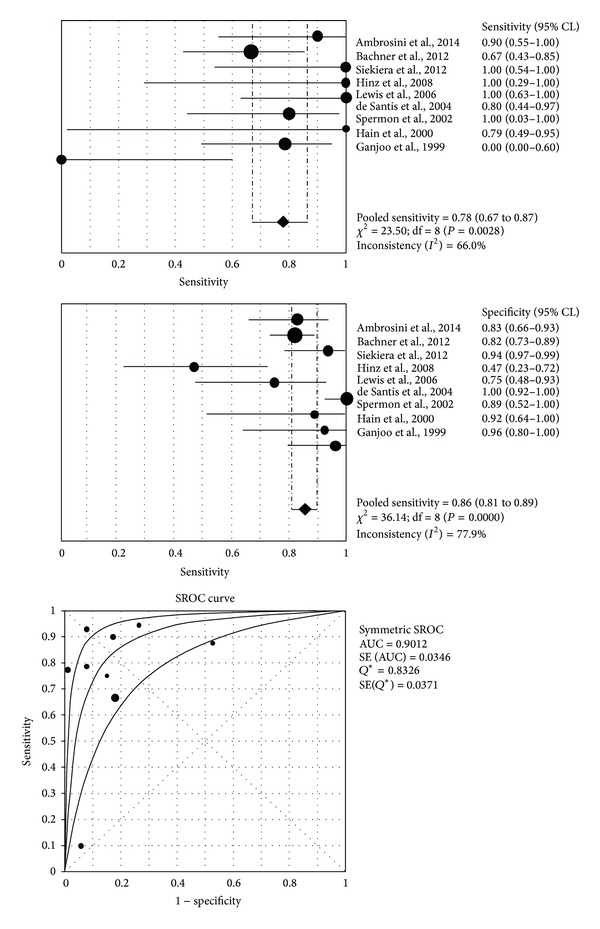
Plots of pooled sensitivity and specificity and summary ROC curve of ^18^F-FDG-PET or PET/CT in the postchemotherapy management of patients with seminoma. The area under the summary ROC curve (0.90) demonstrates that ^18^F-FDG-PET and PET/CT are accurate methods in this setting.

**Figure 3:**
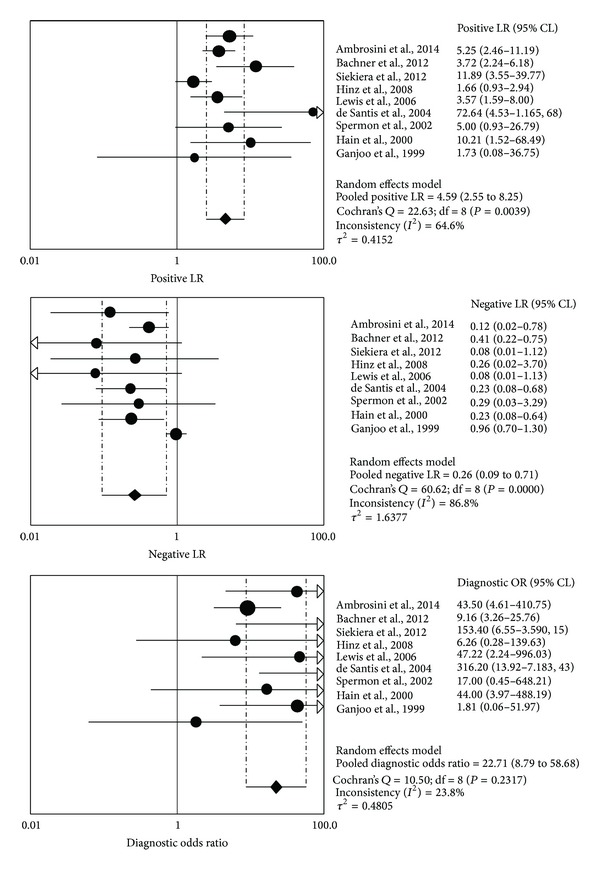
Pooled positive likelihood ratio (LR), negative LR, and diagnostic odd ratio (DOR) of ^18^F-FDG-PET or PET/CT in the postchemotherapy management of patients with seminoma.

**Figure 4:**
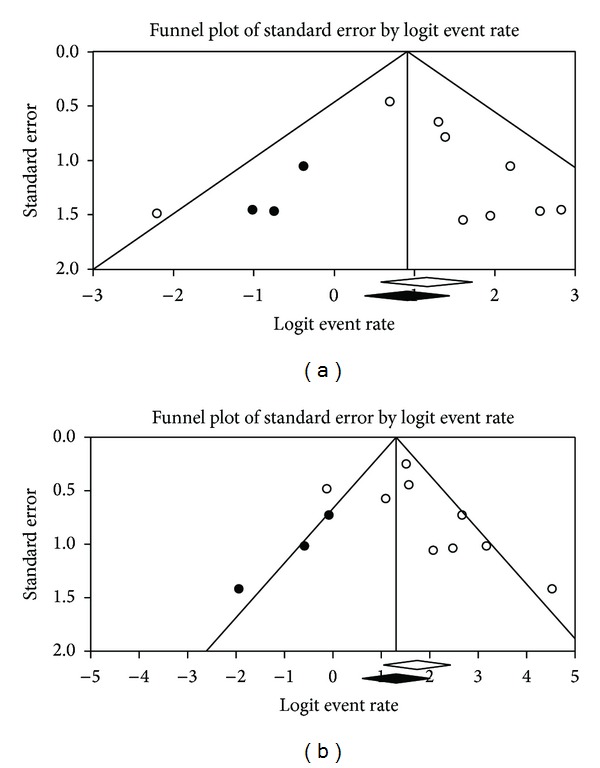
Funnel plots regarding the publication bias on the sensitivity (a) and specificity (b) of ^18^F-FDG-PET or PET/CT in the postchemotherapy management of patients with seminoma.

The diagnostic performance results of ^18^F-FDG-PET or PET/CT in the nine studies selected for the meta-analysis are presented in Figures 2–4 and reported below.

The sensitivity of ^18^F-FDG-PET or PET/CT in the postchemotherapy management of patients with seminoma calculated on a per examination-based analysis ranged from 0% to 100%, with pooled estimate of 78% (95% CI: 67–87%). The specificity of ^18^F-FDG-PET or PET/CT in the postchemotherapy management of patients with seminoma calculated on a per examination-based analysis ranged from 47% to 100%, with pooled estimate of 86% (95% CI: 81–89%). The included studies were statistically heterogeneous in their estimate of sensitivity (*I* ^2^: 66%) and specificity (*I* ^2^: 78%) (Figure 2).

The pooled positive and negative predictive values and accuracy of these methods were 58% (95% CI: 48–68%), 94% (95% CI: 90–96%), and 84% (95% CI: 80–88%), respectively.

The pooled positive LR, negative LR, and DOR were 4.59 (95% CI: 2.6–8.3), 0.26 (95% CI: 0.09–0.71), and 22.7 (95% CI: 8.8–58.7), respectively (Figure 3). The AUC was 0.90 (Figure 2).

Egger's regression intercepts for sensitivity and specificity pooling were 0.7 (95% CI: −1.3 to 2.7; *P* = 0.43) and 1.5 (95% CI: −1 to 4; *P* = 0.19), respectively. Applying Duval and Tweedie's method, the funnel plot of sensitivity and specificity reached symmetry and the adjusted sensitivity and specificity decreased for 3.2% and 8.8%, respectively (Figure 4).

Due to the statistical heterogeneity found in the calculation of the pooled sensitivity and specificity of ^18^F-FDG-PET or PET/CT in the postchemotherapy management of patients with seminoma, subgroup analyses considering residual/recurrent lesions at CT with size > or <3 cm were performed, taking into account data provided by seven out of nine articles included in our meta-analysis. The subgroup analyses demonstrated that the pooled sensitivity and specificity of ^18^F-FDG-PET or PET/CT were 47% (95% CI: 21–73%) and 89% (95% CI: 82–94%), respectively, for patients with postchemotherapy residual/recurrent lesions at CT < 3 cm and 89% (95% CI: 75–97%) and 81% (95% CI: 73–88%), respectively, for patients with postchemotherapy residual/recurrent lesions at CT > 3 cm. Furthermore, the statistical heterogeneity largely decreased (*I* ^2^ was 0% for sensitivity and specificity in both subgroup analyses). The AUC for patients with postchemotherapy residual/recurrent lesions at CT < or >3 cm was 0.76 and 0.87, respectively.

## 4. Discussion

Our systematic review and meta-analysis assessed the diagnostic performance of ^18^F-FDG-PET or PET/CT in the postchemotherapy management of patients with seminoma, including evaluation of residual masses after chemotherapy and restaging. Several studies have used ^18^F-FDG-PET or PET/CT in this setting reporting different values of sensitivity and specificity (Figure 2). However, some of these studies have limited power, analyzing only a relatively small number of patients. In order to derive more robust estimates of the diagnostic performance of ^18^F-FDG-PET or PET/CT in this setting we have pooled published studies [25]. A systematic review process was adopted in ascertaining studies, thereby avoiding selection bias. Furthermore, the quality of the included studies was assessed by using the 2011 Oxford Center for Evidence-Based Medicine checklist for diagnostic studies (Table 4).

A previous meta-analysis in German language evaluated the diagnostic value of ^18^F-FDG-PET in the assessment of residual tumors after systemic treatment of metastatic seminoma including 5 studies only (130 patients) [26]. Conversely, our updated pooled analysis includes 9 articles (375 examination), thus providing more robust results on a statistical point of view.

Pooled results of our analysis indicate that ^18^F-FDG-PET and PET/CT have good sensitivity (78%), specificity (86%), and accuracy (84%) in the postchemotherapy management of patients with seminoma. ^18^F-FDG-PET and PET/CT have a low positive predictive value (58%) but a very high negative predictive value (94%) for this indication. Furthermore, the value of the AUC (0.90) demonstrates that ^18^F-FDG-PET and PET/CT are accurate diagnostic methods in this setting.

Our subgroup analyses considering the size of residual/recurrent lesions at CT after chemotherapy demonstrate that the sensitivity of ^18^F-FDG-PET and PET/CT significantly increases in patients with residual/recurrent lesions >3 cm compared to those with residual/recurrent lesions <3 cm (89% versus 47%, resp.), whereas the specificity slightly decreases (81% versus 89%, resp.). The overall accuracy of ^18^F-FDG-PET and PET/CT is superior in assessing residual/recurrent lesions >3 cm compared to those <3 cm. On the other hand, it should be underlined that ^18^F-FDG-PET may early detect relapse in the postchemotherapy management of seminoma patients without abnormal findings at CT as reported in some articles [6, 13], because functional abnormalities evaluated by ^18^F-FDG-PET may precede morphological changes.

Performing subgroup analyses for different lesion size of residual/recurrent lesions has substantially great merit to show as this parameter basically represents per se a source of heterogeneity among the studies. As a matter of fact, no significant heterogeneity among the studies included in the different subgroup analyses was found. Unfortunately, there were insufficient data to perform subgroup analyses taking into account the different device used (PET/CT versus PET) because only two studies assessed the diagnostic performance of ^18^F-FDG-PET/CT [6, 8]. Nevertheless, a superior diagnostic performance of PET/CT compared to PET alone is expected.

Possible sources of false-negative and false-positive results for postchemotherapy residual/recurrent seminoma at ^18^F-FDG-PET or PET/CT should be kept in mind. False-negative findings may be due to small lesions (with size below the resolution of the method) or with low proliferative activity (and consequently low ^18^F-FDG uptake). On the other hand, the most frequent cause of false-positive findings for postchemotherapy residual/recurrent seminoma at ^18^F-FDG-PET or PET/CT is inflammatory lesions.

The results of our analysis strengthen and support the recommendations reported in international guidelines about the usefulness of ^18^F-FDG-PET or PET/CT in the postchemotherapy management of patients with seminoma [27, 28]. Patients with complete response after chemotherapy do not require further treatment and are followed up. In the case of residual lesions at CT, ^18^F-FDG-PET or PET/CT may be carried out in a minimum of 6 weeks after ending chemotherapy, in order to reduce false-positive results due to inflammation. In lesions >3 cm, ^18^F-FDG-PET or PET/CT is the recommended approach, whereas, in lesions <3 cm, ^18^F-FDG-PET or PET/CT may be considered. Because the pooled negative predictive value is very high, ^18^F-FDG-PET and PET/CT can replace invasive methods in the evaluation of postchemotherapy residual/recurrent lesions in patients with seminoma [27, 28]. In this context, a negative ^18^F-FDG-PET warrants follow-up only avoiding inappropriate subsequent treatment (surgery, chemotherapy, or radiotherapy). In the case of a positive ^18^F-FDG-PET, the possibility of residual seminoma is high, though a false-positive result cannot be excluded as demonstrated by its low positive predictive value.

Possible limitations of our meta-analysis could be the heterogeneity between the included studies, the publication bias, and the low number of the selected studies [25].

Heterogeneity between studies may represent a potential source of bias in a meta-analysis. This heterogeneity is likely to arise through diversity in methodological aspects between different studies (Table 2). The baseline differences among the patients in the included studies, the reference standard used, and the study quality (Table 4) may contribute to the heterogeneity of the results too. In our pooled analysis the included studies were statistically heterogeneous in their estimate of sensitivity and specificity. However, heterogeneity among the studies was not found performing subgroup analyses for different size of residual/recurrent lesions. In order to limit the heterogeneity we decided to include in our pooled analysis only studies which evaluated the postchemotherapy management of patients with seminoma, excluding those which described the role of ^18^F-FDG-PET or PET/CT at initial staging.

Publication bias is a major concern in all meta-analyses as studies reporting significant findings are more likely to be published than those reporting nonsignificant results. Indeed, it is not unusual for small-sized early studies to report a positive relationship that subsequent larger studies fail to replicate. We assessed publication bias in our meta-analysis using qualitative and quantitative methods (Egger's regression and Duval and Tweedie's method). Funnel plots showed an asymmetry for both sensitivity and specificity pooling, and Duval and Tweedie's method also showed the importance of possible publication bias as estimated unbiased pooled sensitivity and specificity decreased considerably compared to the original estimates.

Only nine studies are included in the quantitative analysis and this could limit the statistical power of our meta-analysis. Some of them presented a low sample size (Table 1). Overall the quality of the included studies was moderate (Table 4). Four studies reported the consecutive recruitment of the patients and only three studies reported blind interpretation of the ^18^F-FDG-PET or PET/CT findings, which can introduce interpretation bias (when observers of PET or PET/CT studies had prior knowledge influencing their interpretation of the results). On the other hand, both prospective and multicentric clinical trials are available to this regard.

## 5. Conclusions

^18^F-FDG-PET and PET/CT were demonstrated to be accurate diagnostic imaging methods in the postchemotherapy management of patients with seminoma (in particular in patients with recurrent/residual lesions >3 cm); nevertheless possible sources of false-negative and false-positive results should be kept in mind. The literature focusing on the use of ^18^F-FDG-PET and PET/CT in this setting still remains limited and cost-effectiveness analyses are warranted.

## Figures and Tables

**Table 1:** Basic study and patient characteristics.

Authors	Year	Country	Study design	Mean age (years)	^ 18^F-FDG PET or PET/CT scans	Scans with residual lesion <3 cm	Scans with residual lesion >3 cm
Ambrosini et al. [6]	2014	Italy	Retrospective and monocentric	37.7	45*	NR	NR
Bachner et al. [7]	2012	Several European countries and Canada	Retrospective and multicentric	NR	127	54	73
Siekiera et al. [8]	2012	Poland	Retrospective and monocentric	NR	37	20	17
Hinz et al. [9]	2008	Germany	Prospective and multicentric	42	20	8	12
Lewis et al. [10]	2006	USA	Retrospective and monocentric	NR	24	13	11
De Santis et al. [11]	2004	Austria and Germany	Prospective and multicentric	NR	56	37	19
Spermon et al. [12]	2002	Netherlands	NR and monocentric	30	10	8	2
Hain et al. [13]	2000	UK	Retrospective and monocentric	30	27	NR	NR
Ganjoo et al. [14]	1999	USA	Prospective and monocentric	38	29	8	18

*Only patients with seminoma who underwent PET or PET/CT for restaging or evaluation of postchemotherapy residual lesions were selected; NR: not reported.

**Table 2:** Technical aspects of  ^18^F-FDG PET and PET/CT in the included studies.

Authors	Device	^ 18^F-FDG mean injected dose	Time between ^18^F-FDG injection and image acquisition	Image analysis
Ambrosini et al. [6]	PET/CT	3–5.7 MBq/kg	60 min	Visual
Bachner et al. [7]	PET	NR	NR	Visual
Siekiera et al. [8]	PET/CT	NR	NR	Visual
Hinz et al. [9]	PET	NR	45–60 min	Visual and semiquantitative
Lewis et al. [10]	PET	NR	NR	Visual
De Santis et al. [11]	PET	370 MBq	>45 min	Visual
Spermon et al. [12]	PET	200–220 MBq	60 min	Visual and semiquantitative
Hain et al. [13]	PET	320 MBq	NR	Visual
Ganjoo et al. [14]	PET	370 MBq	60 min	Visual and semiquantitative

NR: not reported; PET: positron emission tomography; CT: computed tomography.

**Table 3:** Diagnostic accuracy data of  ^18^F-FDG PET and PET/CT on a per examination-based analysis.

Author	Posttherapy evaluation, surveillance, or restaging				Subgroup analyses about recurrent/residual masses at CT							
					Lesions < 3 cm				Lesions > 3 cm			
	TP	FP	FN	TN	TP	FP	FN	TN	TP	FP	FN	TN
Ambrosini et al. [6]	9	6	1	29	NR	NR	NR	NR	NR	NR	NR	NR
Bachner et al. [7]	14	19	7	87	3	8	4	39	11	11	3	48
Siekiera et al. [8]	6	2	0	29	0	1	0	19	6	1	0	8
Hinz et al. [9]	3	9	0	8	2	3	0	3	1	6	0	5
Lewis et al. [10]	8	4	0	12	1	2	0	10	7	2	0	2
De Santis et al. [11]	8	0	2	46	1	0	2	34	7	0	0	12
Spermon et al. [12]	1	1	0	8	0	0	0	8	1	1	0	0
Hain et al. [13]	11	1	3	12	NR	NR	NR	NR	NR	NR	NR	NR
Ganjoo et al. [14]	0	1	4	24	0	1	2	5	0	0	1	17

NR: not reported; TP: true positive; FP: false positive; FN: false negative; TN: true negative.

**Table 4:** Quality assessment of the included studies.

First author/year	Spectrum of patients (only information regarding seminoma patients were included)	Consecutive or random selection of patients	Reference standard	Application of reference standard regardless of index test	Enough explanation of the index test to ensure reproducibility	Independent blind comparison between index test and reference standard
Ganjoo, 1999 [14]	Seminoma patients after primary or salvage chemotherapy	N/A	Follow-up including chest radiograph, tumor markers, physical examination, and CT	Yes	Yes	N/A

Hain, 2000 [13]	13 seminoma patients with possible relapse	Yes	Histology or clinical follow-up (minimum of 18 months)	Yes	Yes	N/A

Spermon, 2002 [12]	10 seminoma patients after completion of first-line chemotherapy	N/A	Histology in 2 patients and clinical follow-up in 8 patients (serum tumour markers, CT, and the duration of the event-free follow-up) (median of 12 months)	Yes	Yes	Yes

De Santis, 2004 [11]	Patients with metastatic pure seminoma who had radiographically defined postchemotherapy residual masses	N/A	Histology (11 resected masses) or follow-up (median of 34 months)	Yes	Yes	N/A

Lewis, 2006 [10]	Patients with residual mass after primary (14 patients) or salvage chemotherapy (10 patients)	N/A	Histology or clinical follow-up	Yes	No	N/A

Hinz, 2008 [9]	Patients with residual (18 patients) or recurrent (2 patients) after chemotherapy	Yes	Histology	Yes	Yes	Yes (though CT information was available to the readers)

Bachner, 2012 [7]	Patients with metastatic seminoma and residual masses after chemotherapy	Yes	Histology or follow-up (at least 24 months)	Yes	Yes	Yes

Siekiera, 2012 [8]	Patients with advanced seminoma after chemotherapy or radiotherapy in their follow-up scheme	N/A	Histology (7 patients) or follow-up	Yes	No	N/A

Ambrosini, 2014 [6]	Seminoma patients for restaging (16 patients), routine follow-up (18 patients), or suspected relapse (10 patients).	Yes (all patients in a PET center)	Histology or follow-up	No (the treatment of the patients was affected by the PET results)	Yes	N/A
